# Empowering Diabetics: Advancements in Smartphone-Based Food Classification, Volume Measurement, and Nutritional Estimation †

**DOI:** 10.3390/s24134089

**Published:** 2024-06-24

**Authors:** Afnan Ahmed Crystal, Maria Valero, Valentina Nino, Katherine H. Ingram

**Affiliations:** 1 Department of Computer Science, Kennesaw State University, Kennesaw, GA 30060, USA; acrystal@students.kennesaw.edu; 2 Department of Information Technology, Kennesaw State University, Kennesaw, GA 30060, USA; 3 Departement of Industrial and Systems Engineering, Kennesaw State University, Kennesaw, GA 30060, USA; lvallad1@kennesaw.edu; 4 Department of Exercise Science and Sport Management, Kennesaw State University, Kennesaw, GA 30060, USA; kingra14@kennesaw.edu

**Keywords:** diabetes, food image recognition, convolutional neural networks, mobile vision, glucose monitoring

## Abstract

Diabetes has emerged as a worldwide health crisis, affecting approximately 537 million adults. Maintaining blood glucose requires careful observation of diet, physical activity, and adherence to medications if necessary. Diet monitoring historically involves keeping food diaries; however, this process can be labor-intensive, and recollection of food items may introduce errors. Automated technologies such as food image recognition systems (FIRS) can make use of computer vision and mobile cameras to reduce the burden of keeping diaries and improve diet tracking. These tools provide various levels of diet analysis, and some offer further suggestions for improving the nutritional quality of meals. The current study is a systematic review of mobile computer vision-based approaches for food classification, volume estimation, and nutrient estimation. Relevant articles published over the last two decades are evaluated, and both future directions and issues related to FIRS are explored.

## 1. Introduction

Diabetes is a chronic metabolic condition that impacts the body’s use of food for energy. The body transforms carbohydrates from food into glucose; when subsequently released into the bloodstream, this is often referred to as “blood sugar”. Rising blood glucose levels trigger the release of the hormone insulin from the pancreas. Insulin acts by allowing glucose to enter the cells, where it can be used for energy or stored for future use. Diabetes is a serious condition in which glucose is unable to enter the cells, either because the pancreas does not secrete insulin sufficiently or insulin does not appropriately trigger the uptake of glucose. If diabetes is not carefully managed, it may lead to severe health problems such as vision loss, heart disease, and kidney disease. Diabetes does not yet have a cure, but healthy management of the condition includes reducing sugar intake, regular exercise, and/or pharmaceutical intervention [1,2].

Diet monitoring is an important strategy for managing blood glucose levels in people living with diabetes [3]. However, food logs or diaries can be difficult to complete with accuracy. Dietary information is easily forgotten or even purposefully omitted. Several automated techniques have been introduced to simplify diet tracking, though all contain various limitations.

Modern imaging technologies, such as wearable technology, tablets, and smartphones, have been created to improve the accuracy of diet monitoring. Smartphone technology that can accurately classify foods and calculate calories has been developed for consumer use [4]. Picture-based applications for nutrition assessment are now possible thanks to recent advances in the processing capabilities of smartphones. Food image recognition systems (FIRS) use real-time food images captured by a camera device as inputs. To identify which food class the image belongs to, the model can utilize multiple tuned hyperparameters. Automated food picture retrieval and categorization has been shown to improve the objectivity of dietary monitoring [5].

### 1.1. Contribution

Our paper presents an overview of recently published automated food intake monitoring systems, with a focus on applications including image analysis specifically developed for managing diabetes. The main contributions of this paper are:

- A systematic review of current research on FIRS and its potential for assisting in the management of diabetes, including limitations and future research directions
- Detailed information on the implementation of FIRS and dietary assessment systems based on reviewed papers, emphasizing how these systems can be integrated into the assessment of diabetic diets
- Examination of unresolved issues that would enhance the performance of current FIRS for improved nutrition analysis in the context of bettering diabetic health

### 1.2. Paper Organization

The rest of the paper is structured as follows: Section 2 explains the research design and methodology systematically used to write this paper, and puts forth prospective research questions on the aspect of food image recognition/classification; Section 3 provides a thorough overview of existing FIRS along with their performance and architectures; Section 4 discusses findings, insights, challenges, and future research directions; finally, Section 5 concludes the paper.

## 2. Research Design and Methodology

### 2.1. Research Goals

In this study, we explore the following research questions:

- RQ1: What cutting-edge technologies are being applied to the classification of food images and macronutrients/carbohydrate intake/blood glucose spikes from consumed foods?
- RQ2: What are the technical challenges of current machine learning (ML) processes and datasets faced by researchers in this domain?
- RQ3: What future work might take place to advance food detection in a more efficient manner, and how may it help diabetic patients?

### 2.2. Primary Studies Selection

This systematic review includes peer-reviewed papers published between 2007 and May 2023 containing the following specific keywords and Boolean operators.

- “Food Image Recognition”
- “Food Image Classification CNN”
- “Food Image Classification Nutrition Analysis”
- “Food Recognition Technology Health Applications”
- “Image Based Food Recognition”
- “Image Based Food Recognition Dietary Assessment” and
- “Food Image Recognition Diabetes”

The search was performed using the following databases.

- “Google Scholar”
- “IEEE Xplore Digital Library”
- “PubMed”
- “ACM Digital Library”
- “ScienceDirect” and
- “Scopus”

### 2.3. Search Procedure and Results

**Figure 1:**
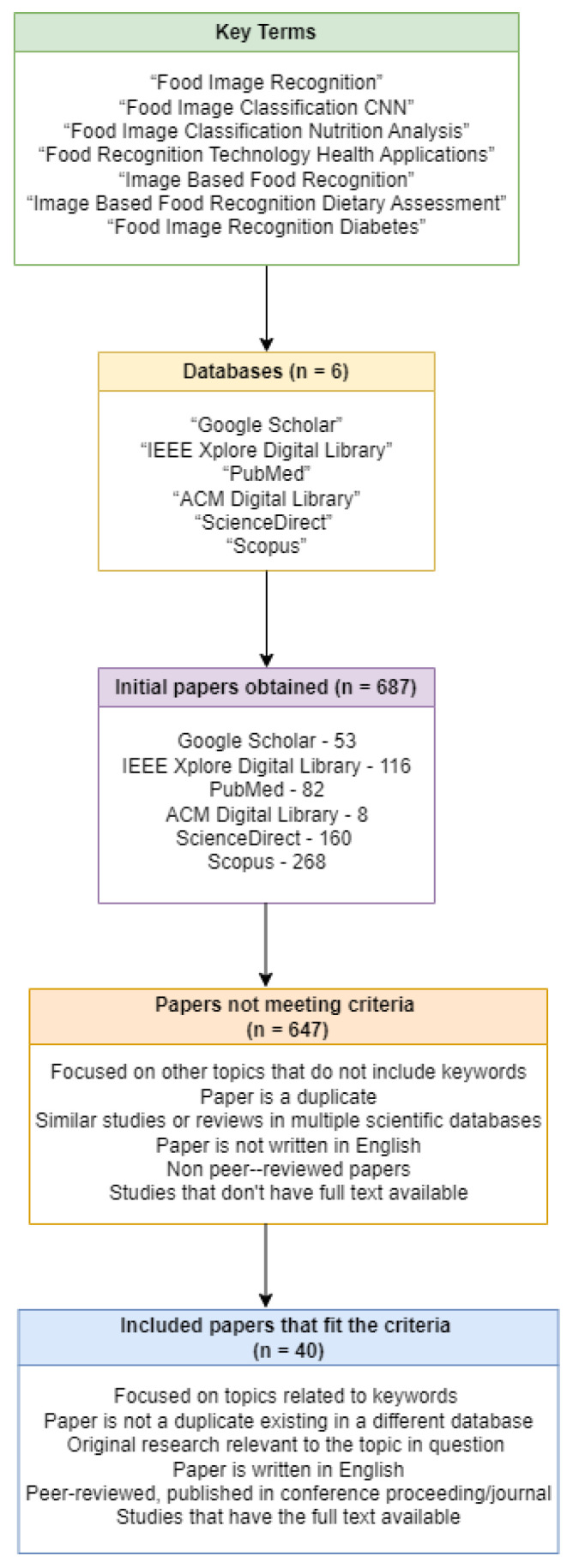
Filtration procedure for systematic literature selection.

The initial search yielded 910 papers in Scopus, and was further refined to include only peer-reviewed articles, conference papers, review papers, and conference review papers. Additional keywords were then applied to limit the articles, including: Deep Learning, Convolutional Neural Networks, Convolutional Neural Network, Image Processing, Image Classification, Machine Learning, Computer Vision, Deep Neural Networks, Neural Networks, Image Recognition, Transfer Learning, CNN, and Food Classification. The source type was limited to journals and conference proceedings. Only papers published in English were included. After this, the results were narrowed to 268 papers. The databases are displayed in Figure 1.

### 2.4. Inclusion and Exclusion Criteria

To refine the list of research papers for our review, we conducted an inclusion and exclusion process using the criteria delineated in Table 1. After removing duplicate studies, the papers were further narrowed by title and abstract, then by a full-text review of the results and conclusions. The screening process ultimately identified 40 papers that aligned with our goal of identifying papers detailing mobile applications that use a food image recognition system (FIRS) and/or other diet/nutrition analysis tool. Several among these were specific to managing blood glucose concentration. The selected papers addressed food image classification and nutrition/blood glucose prediction, providing insights relevant to our research. We further identified studies that utilized food image recognition for dietary management of blood glucose. An overview of these is shown in Table 2.

## 3. Mechanisms of Various FIRS

### 3.1. Basic Architecture

**Figure 2:**
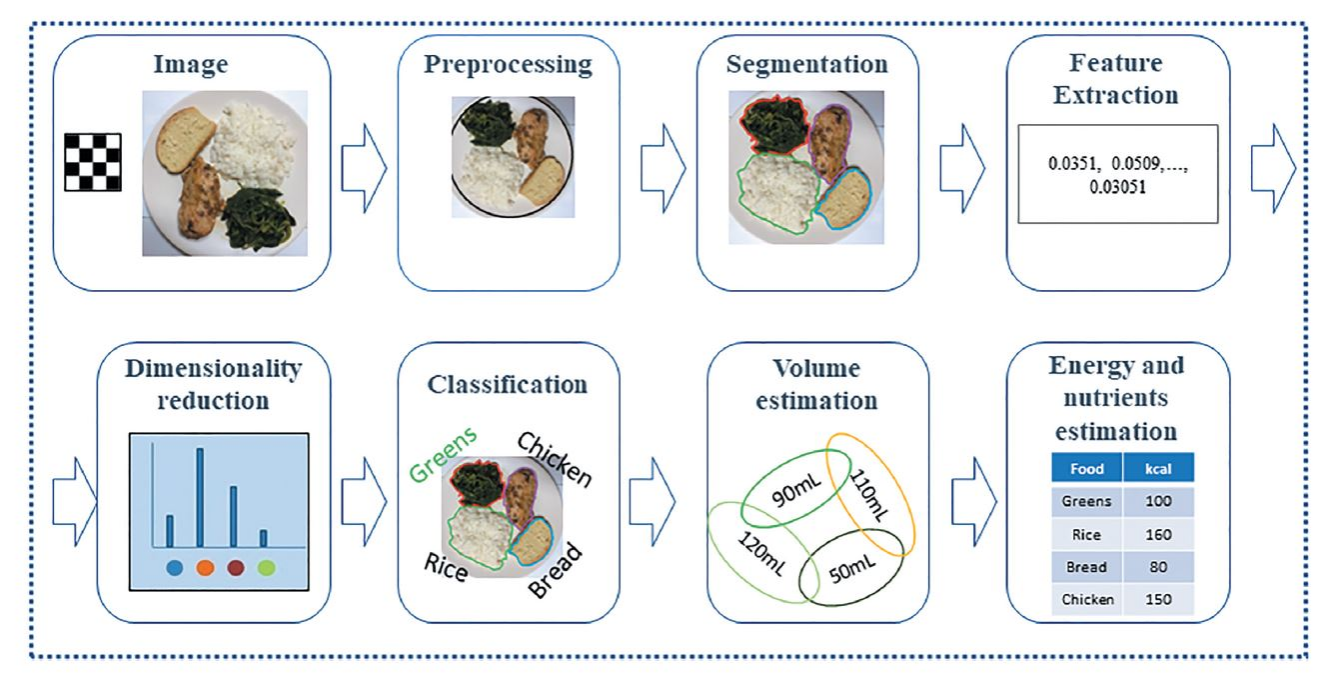
Architecture of FIRS for dietary assessment, as illustrated by [13].

**Figure 3:**
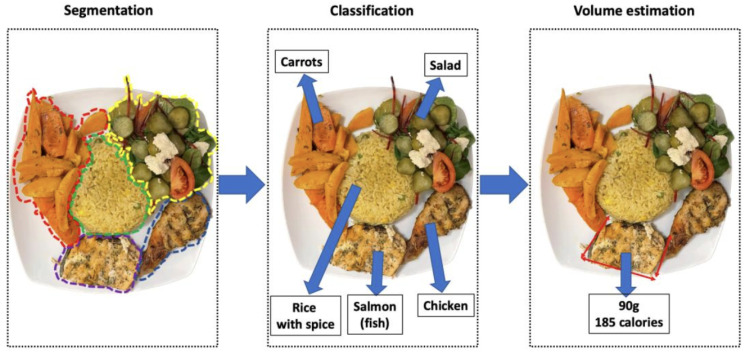
Schematic workflow of current food segmentation, classification, volume, and calorific estimation methods, as illustrated by [14].

Figure 2 illustrates the basic system architecture of mobile-based nutritional monitoring systems [13], while Figure 3 depicts the segmentation-to-volume estimation phase [14]. Table 3 provides a detailed explanation of the process. These systems function as follows: users capture a picture of their meal using a smartphone camera; the image undergoes preprocessing, and additional information can be input if required; next, the different food or beverage categories are separated into distinct locations through segmentation; feature extraction and classification techniques are applied [15]; finally, the estimated calorie content or nutritional value of the meal is calculated [16] and the volume of each food region is computed using nutritional databases [17]. For example, Ravi et al. developed a smartphone application that classifies food intake and provides real-time feedback to users [18].

Conventional classification systems typically involve three sequential activities: segmentation, feature extraction, and classification.

Food classification techniques can primarily be categorized into deep learning (DL)-based recognition and manual feature-based recognition. Examples of deep learning architectures include:

- AlexNet: 1000 distinct classifications were created using a massive deep convolutional neural network to categorize 1.2 million high-resolution photos. It significantly outperformed the prior state-of-the-art, with a top-5 error rate of 15.3% on test data [19].
- VGG: VGG16 contains a total of over 138 million parameters, making it a fairly large network [20,21].
- ResNet: The family of very deep architectures known as deep residual networks exhibits remarkable convergence patterns and impressive accuracy [22].
- Inception: On the ImageNet dataset, the Inception v3 image recognition model has demonstrated accuracy levels of above 78.1%. The model consists of convolutions, average pooling, max pooling, concatenations, dropouts, and fully linked layers, among other symmetric and asymmetric building pieces [23,24].
- GoogLeNet: Convolutional neural networks of the GoogLeNet type are built on the Inception design. Through the use of Inception modules, the network is able to select among a variety of convolutional filter sizes for each block [25].

Examples of machine learning algorithms include:

- SVM: A supervised machine learning approach called Support Vector Machine (SVM) is utilized for classification. The SVM algorithm’s primary goal is to locate the best hyperplane in an N-dimensional space that may be used to divide data points into various feature space classes [26].
- KNN: A non-parametric supervised learning classifier, the k-nearest neighbors (KNN) algorithm uses proximity to classify or predict how a single data point will be grouped [27].
- Random Forest (RF): A popular machine learning approach, this aggregates the output of several decision trees to obtain a single outcome [28].
- Naïve Bayes: The Naïve Bayes classifier is an algorithm for supervised machine learning that applies probability principles to classification tasks. As a member of the generative learning algorithm family, it aims to simulate the input distribution of a certain class or category [29].

Traditional machine learning classifiers are used to determine the type of each segmented area based on handcrafted features [30]. Convolutional neural networks (CNNs) are commonly used for feature extraction, where machine learning classifiers or CNN layers are used for classification [31]. Dimensionality reduction techniques may be employed to enhance accuracy by selecting only the most important features [32].

### 3.2. Sensing Mechanism

The majority of studies in this review utilize a picture taken by a mobile camera [4]. Because mobile devices such as a smartphones or tablet are quite portable, have better functionality compared to their non-mobile equivalents, and are capable of producing quickly shareable crystal-clear images comparable to professional high-resolution cameras, they are ideal for capturing raw images for use in training food image classification and volume estimation models for the purpose of helping diabetics with diet monitoring. The existence of a digital camera and high computational ability in a single device allow for swift processing of photos before displaying them to the user. In theory, conventional image processing techniques can be used to process images that are taken with mobile devices; however, when used directly such algorithms are less effective due to various limitations and other factors. Compared to computers made expressly for processing images and videos, the majority of mobile devices still have lower processing capability. They are also battery-operated; in the case of a mobile phone, its connectivity and communication are the main functions that require conservation of the battery’s limited energy. Particularly with smartphones, there are new difficulties for mobile image processing; for instance, due to market competitiveness, cellphones now have cameras with very high resolutions and large pixel counts, which necessitates the use of more effective algorithms. The resolution of sensors in mobile devices has grown rapidly in recent times; unfortunately, form factor limitations have made it challenging to enhance mobile camera optics. To address this issue, smartphones now come equipped with multiple cameras pointing in the same direction [33]. To address this, several studies have used pictures taken from multiple camera angles [34] or photographs taken from various databases.

### 3.3. Data Preprocessing

Preprocessing input images can improve the representation of food photos used for classification by addressing issues such as lighting conditions, orientation, color distribution, distortion, and blur [35]. Minor standard editing techniques such as contrast equalization, scaling, and cropping can enhance the original image. Additionally, fundamental image processing techniques, including rotation, cropping, noise addition, and manipulation of features such as contrast, saturation, brightness, and hue, can generate more food photos from existing datasets, which is beneficial in cases where the dataset is small.

### 3.4. Manually Designed Features Approach

Feature extraction refers to finding the descriptor or feature vector that most effectively reveals underlying visual information. Feature extraction is carried out manually by examining simple visual characteristics such as color, texture, and shape present in food photographs. Detailed visual data can be extracted using a number of widely used feature extraction techniques, including Scale-Invariant Feature Transform (SIFT), Histogram of Oriented Gradients (HOG), Gabor filter, MR8 filter, and Local Binary Patterns (LBP). More complex feature descriptors can be built by fusing various feature vectors to increase the accuracy of food classification. Table 4 provides an overview of publications that utilize manual feature extraction.

Chokr et al. extracted unprocessed attributes from food photos using the Mathworks Image Processing Toolbox. Following the use of InfoGain and principal component analysis on the initial 6000 features in place of the total 11,868 features, they managed to characterize each food image using 23 visual attributes [36].

Zhang et al. used a linear SVM classifier to create the Snap-n-Eat smartphone app for food recognition. To divide the food photos into various segments comprising varying foods, the authors used saliency and region-based sampling. Low-level characteristics were retrieved from each region and then utilized by the SVM classifier to identify food categories. This classifier identified fifteen different food categories with an accuracy rate of above 85% [37].

On the other hand, Silva and colleagues used a quadratic SVM on an artificially augmented database with 60 food classes to create an interactive mobile app for automatic food detection. On the Food-101 dataset, their SVM classifier using histogram of oriented gradients (HOG), color, Gabor filter, modified local binary patterns (LBP), and sped-up robust features (SURF) surpassed conventional methods utilizing handcrafted features [38].

Kong et al. used pictures captured from various angles to overcome occlusion in the images. This process generated descriptors built upon the Scale-Invariant Feature Transform (SIFT) and Difference of Gaussian (DoG) techniques. After the descriptors were delineated, a nearest neighbor classifier was used for food classification [39]. This approach was later expanded to improve the SIFT descriptions. The DietCam system by Kong et al. is an automated multi-view food classifier designed for use in a health monitoring system. It calculates SIFT descriptors, using three separate food photos or a short video around the meal to realize 3D reconstruction. As employing several hundred SIFT characteristics to compare the similarity of images is computationally expensive, the authors suggested employing an effective hierarchical k-means clustering method to group SIFT characteristics into visual words [40].

**Figure 4:**
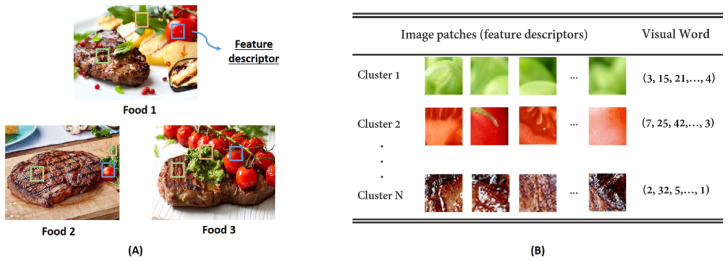
(**A**) Feature point extraction and (**B**) visual word construction–in this case, pesto steak with balsamic tomatoes [41].

**Figure 5:**
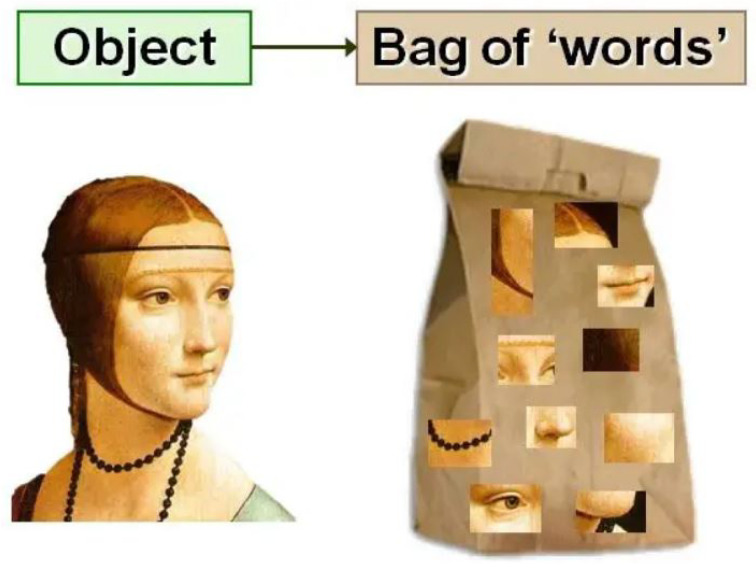
Image classification using the bag of visual words technique [42].

Visual words refers to the most representative description among a given group of descriptors in the same cluster. Pictorial descriptions of feature point extraction and visual word construction are provided in Figure 4 [41]. Another example using a painting is also provided in Figure 5, courtesy of [42]. These visual words may be used as a foundation for a visual library. Other coding methods can be used to increase efficiency in addition to visual language.

Anthimopoulos et al. used a Bag-of-Features (BoF) approach to demonstrate that adding more visual words could further increase identification accuracy of foods, with a categorization accuracy of around 78%. They further demonstrated that among the SIFT-based and color-based approaches, hsvSIFT and color moment invariants along with opponent color histogram had the best performance. Numerous linear and nonlinear methods were also assessed, including linear SVM, RBF SVMs, ANNs, and Random Forest (RF). While the dataset used in this study was large (around 5000 images), the small geographical location used may present a limitation in generalizing the findings, as the study was restricted to central European cuisine(s) only [6].

The Fisher Vector (FV) is used to encode features instead of directly inputting them to classifiers, which speeds up picture recognition. Beijbom et al. extracted five different sorts of features, comprising HOG, color, local binary patterns (LBP), SIFT, and filter responses from the MR8 filter bank. Using a dictionary learned via k-means clustering, these attributes were encoded using Locality-constrained Linear Coding (LLC) [43].

**Table 4:** Articles using manual feature extraction techniques for food image recognition or nutrition evaluation.

Author(s)	Application	Dataset	Methods	Accuracy	Year
Puri et al. [34]	Food Recognition and Volume Estimation	400 image sets with 150 varieties of food, 11 classes	Adaboost-based feature selection	around 90%	2009
Chen et al. [44]	Food Recognition and Quantity Estimation	50 categories with 100 images in each	SIFT, Local binary pattern feature descriptors, Gabor filter descriptors, and color histograms for feature extraction; multi-label SVM classifier and AdaBoost	68.3% overall	2012
Hoashi et al. [45]	Food Image Recognition	85 food groups	Multiple kernel learning combined with gradient histogram, color histogram, bag-of-features (BoF), and gabor features	62.52%	2010
Kawano et al. [46]	Food Recognition	6781 food images	Bounding boxes, GrabCut, color histogram, SURF-based bag-of-features, linear SVM and fast 2 kernel	81.55% for top 5 category candidates	2013
Chokr et al. [36]	Calories Prediction from Food Images	over 1000 food images in six different categories	Mathworks Image Processing Toolbox, Principal Component Analysis (PCA), Naive Bayes, Regularized Linear Regression, Logistic Regression, Multilayer Perceptron, Random Forests and Support Vector Machines (SMO version)	99.1%	2017
Zhang et al. [37]	Food Recognition and Nutrition Estimation	2000 images in 15 food categories	SVM, hierarchical grouping algorithm, Saliency-Based Sampling, Region-Based Sampling, Fisher vector representation, stochastic gradient descent	above 85%	2015
Silva et al. [38]	Diet Monitoring System	60 food classes with 1000 images each	Quadratic SVM, Color, HOG, modified LBP and Gabor	65.5%	2018
Kong et al. [39]	Food Recognition	Pittsburgh Food Image Dataset (PFID), 61 categories	Gaussian region detector, Scale Invariant Feature Transform (SIFT), color histogram, SVM, nearest neighbor classifier	84%	2011
Tammachat et al. [47]	Calories Analysis of Food Intake	40 types of Thai food, 100 images for each	SVM, Bag-of-Features (BOF), Segmentation-based Fractal Texture Analysis (SFTA), Color Histogram	about 70%	2014
Beijbom et al. [43]	Food Logging from Images	Menu-Match dataset, 1386 tagged food items in 646 images across 41 categories	color, histogram of oriented gradients (HOG), scale-invariant feature transforms (SIFT), local binary patterns (LBP), and filter responses from the MR8 filter bank, locality-constrained linear encoding (LLC), k-means clustering, linear SVM	77.4%	2015
Kawano et al. [48]	Food Image Recognition	UEC-FOOD100	Fisher Vectors, SVM, Color patches, RootHoG patches	72.26%	2014
He et al. [49]	Multiview Food Recognition	15,262 food images of 55 food types	Multiview multikernel SVM, RGB 3-D histogram	90%	2015

While hand-engineering features is time consuming and fragile, deep learning has emerged as a preferred technique for extracting discriminative feature representations from unstructured data such as food photographs and videos. Overall, using deep learning networks has been shown to be more robust and resilient compared to using hand-crafted features, leading to their adoption in recent algorithms for food vision analysis.

### 3.5. Deep Learning End-to-End Method

Convolutional Neural Networks (CNNs) are a deep learning (DL) approach that works very well for tasks involving picture recognition. DL-based approaches have gained popularity in food recognition thanks to their ability to automatically learn crucial traits necessary for classifying food classes [50,51]. CNN, Deep CNN, InceptionV3, and ensemble algorithms are common DL-based methods for identifying food in images [52,53,54,55]. Table 5 provides an overview of publications that have utilized deep learning for food image recognition.

Liu et al. proposed DeepFood, a food recognition and dietary evaluation system using a DL model to research and evaluate food items photographed by smartphones. They suggested an approach that uses a pre-trained and fine-tuned CNN for object classification and region detection to recognize numerous food photos. In order to categorize and locate each image region in the original photos, each image region is mapped into a feature map. The accuracy obtained for Food 101, UEC-100, and UEC FOOD 256 was 77.4%, 76.3%, and 54.7%, respectively [56].

Ma et al. fine-tuned the Inception V3 architecture to train their model. In all, 10,074 pictures spanning 100 culinary categories were gathered [16]. The nutritional assessment mostly relies on the classification outcome, as the food category is already determined by a set nutritional configuration (e.g., calories, carbohydrates, proteins). Additionally, they performed nearly identical tests on a different dataset, ChinaMartFood-109 [57].

Akti et al. concentrated on creating a mobile-friendly Middle Eastern food identification application using the MobilenetV2 deep learning model. They used data augmentation techniques on the dataset to compensate for the lack of data in certain food classes. Their experimental findings demonstrated the accuracy and memory management advantages of adopting the MobilenetV2 architecture for food recognition. This tool help blind users by automatically recognizing foods from 23 different food classes with 94% accuracy [58].

For Chinese food recognition systems, research by Teng et al. suggested a compact and effective convolutional neural network architecture based on Bag-of-Features. The suggested architecture was optimized using backpropagation, which is essential for accurate recognition. The proposed architecture and conventional Bag of Features model were compared and correlated in order to find similarities and the pinpoint elements affecting recognition accuracy [59].

Tai et al. used transfer learning and the EfficientNet architecture to create a novel dish recognition system. They adjusted EfficientNet-B0 by adding a number of significant layers and retraining the model on the UEH-VDR dataset, which consists of images of different Vietnamese dishes. They then used transfer learning to make use of the optimal parameters discovered by pretraining the model on the ImageNet dataset [60].

Both the status and kind of food items are simultaneously recognized in a study proposed by Alahmari et al., who used a novel cascaded multi-head technique based on deep learning. On a benchmark dataset of food ingredient photos with nine varying food states and eighteen different food types, they trained and assessed the suggested methodology alongside contrasting the suggested method with a deep learning method that was not cascaded. The cascaded methodology was able to perform food ingredient state recognition with an accuracy of 87%, compared to 81% when using the non-cascaded DL approach [61].

Lubura et al. assessed Serbian food waste generation using before and after photographs of 20–30 year old adults’ daily meals in the region. A CNN was used to estimate the amount of food waste based on clicked photos. An image dataset comprising 157 different food categories was created by gathering the most popular food item photographs from the internet. The database contained a total of 23,552 photographs, with each category including between 50 and 200 pictures. The study reported that the average amount of food wasted per meal in Serbia throughout the analysis period was 21.3% [62].

Fakhrou et al. suggested a smartphone-based system for fruit and dish recognition using a deep CNN model. They combined two CNN architectures to create a new model for food recognition using ensemble learning methodology [63].

Liu et al. employed the EfficientDet deep learning model for the creation of a multiple-dish identification model. The model was created using three different meal types from 87 different varieties of regional Taiwanese cuisine [64].

**Table 5:** Deep learning techniques used in food image recognition and/or nutrition evaluation publications.

Author(s)	Application	Dataset	Methods	Accuracy	Year
Qayyum et al. [52]	Food Image Prediction	≈5000 images	CNN	82.03%	2018
Tomescu et al. [53]	Food Recognition	80,000 images and 382 food classes taken from Google Images	EfficientNets (EfficientNet-B0)	70.3%	2020
Cornejo et al. [54]	Food Image Recognition	3600 food images	ResNet-50	99.9%	2021
Tahir et al. [55]	Food Image Analysis	Food101, UECFOOD100, UECFOOD256, Malaysian Food dataset of 775 classes	MobileNetV3, ImageNet	99.12%	2021
Bossard et al. [65]	Food Image Recognition	Food-101	CNN, Random Forests (RF) Component Mining	56.40%, 50.76%	2014
Mezgec et al. [66]	Dietary assessment of Parkinson’s disease patients	520 different food and drink items	Modification of AlexNet	86.72%	2017
Christodoulidis et al. [67]	Food Recognition	Manually annotated dataset with 573 food items	6-layer deep convolutional neural network	84.9%	2015
Hassannejad et al. [68]	Food Recognition	ETH Food–101, UEC FOOD 100, UEC FOOD 256	Inception V3	88.28%, 81.45%, and 76.17%, respectively	2016
Liu et al. [56]	Food Image Recognition	Food 101, UEC–100, UEC FOOD 256	CNN, ImageNet	77.4%, 76.3%, and 54.7%	2016
Park et al. [69]	Korean food image detection	92,000 Korean food images categorized into 23 groups	Deep Convolutional Neural Network (DCNN)	91.3%	2019
Ma et al. [16]	Image-based nutrient estimation	10,074 pictures spanning 100 cuisine categories	CNN, Inception V3	78.26%	2021
Akti et al. [58]	Middle Eastern food identification	5723 images in 23 food classes	Mobilenet–V2 deep learning model	94%	2022
Teng et al. [59]	Recognition of Chinese food	8734 images in 25 different categories	5-layer deep convolutional neural network	97.12%	2019
Tai et al. [60]	Dish Recognition	UEH–VDR	EfficientNet–B0	92.33%	2022
Alahmari et al. [61]	Food State Recognition	Training, validation, test sets—5251, 1132, 1180 images respectively	CNN	86.69%	2022
Zhang et al. [70]	Food Recognition	1455 food pictures, 612 high–resolution food images	Deep CNN	89.60%	2022
Lubura et al. [62]	Food Recognition	23,552 images covering 157 food categories	CNN	98.8%	2022
Ciocca et al. [71]	Classification of food images	475 food classes and 247,636 photos	CNN based on the Residual Network with 50 layers architecture	81.59%	2018
Yanai et al. [72]	Food image recognition	FOOD100 and UEC-FOOD256	DCNN, ImageNet	78.77% and 67.57% respectively	2015
Fakhrou et al. [63]	Fruit and dish recognition	31,127 images classified into 29 different classes	Deep CNN model	95.55%	2018
Liu et al. [64]	Taiwanese Multi-Dish Food Recognition	87 types of Taiwanese dishes among 4733 images	EfficientDet deep learning	80%, mean average precision (mAP) 0.92%	2018

These studies demonstrate the effectiveness and applicability of DL techniques for food recognition and highlight the advantages of DL models over traditional methods in terms of recognition accuracy.

Effective food classification models can help diabetic patients to automate their diet tracking process and greatly reduce the tediousness of related tasks, as patients do not have to manually record food logs when using the system, potentially improving their quality of life.

### 3.6. Segmentation

Anthimopoulos et al. [73] proposed the use of the Canny detector to locate dining plates in an image with precision, along with a segmentation method that transforms the image into the CIELAB color space. The resulting fine-grained texture is smudge-free, and the dominant color edge is retained using pyramidal mean shift filtering.

**Figure 6:**
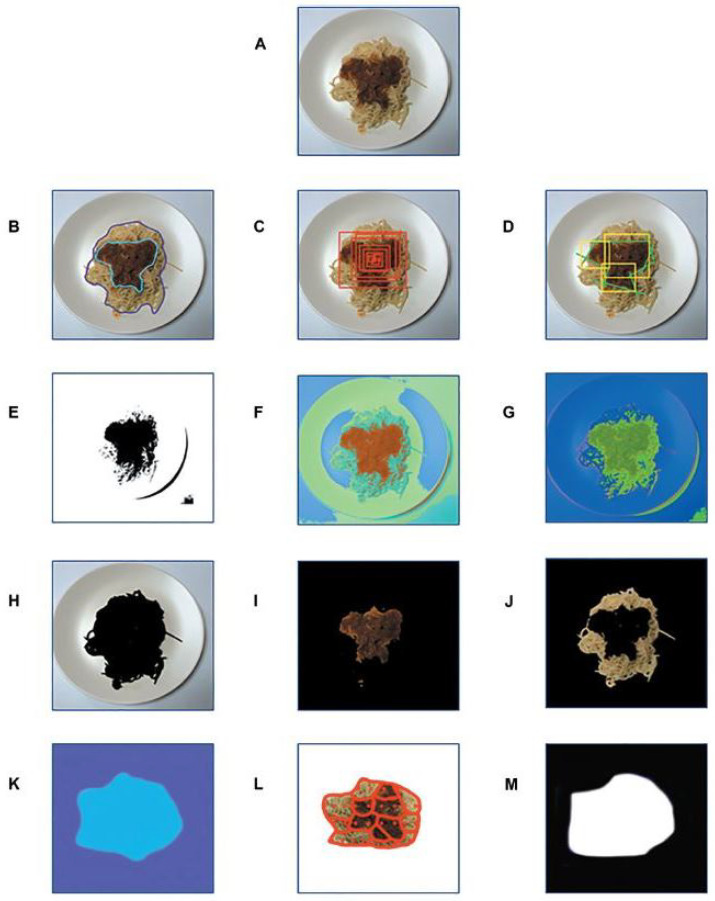
Various segmentation methods: (**A**) Initial image of plate with user’s meal. (**B**) Manual segmentation of initial image. The user draws a line/border/polygon manually around each food item. (**C**) Hierarchical segmentation of initial image. (**D**) Saliency-aware segmentation of initial image. (**E**) Thresholding segmentation of initial image. (**F**) Clustering segmentation of initial image. (**G**) Segmentation of initial image based on Sobel operator. (**H**) Color/texture-based segmentation of initial image. (**I**) Color/texture-based segmentation of initial image. The second cluster depicts the sauce. (**J**) Color/texture-based segmentation of initial image. The third cluster depicts the spaghetti. (**K**) Thermal clustering of initial image. (**L**) Region-based segmentation of initial image. (**M**) Segmentation based on CNNs on initial image [13].

A description of segmentation techniques used in image-based food recognition systems (shown in Figure 6) is presented below [13]:

(A) Manual segmentation: Users draw lines, borders, or polygons around each food item.
(B) Hierarchical segmentation: Over-segmentation is performed initially, followed by finer-grained segmentation based on a criterion.
(C) Saliency-based segmentation: Highlights food regions and suppresses non-food regions using spatial, color, and statistical characteristics.
(D) Threshold-based segmentation: Produces a binary image by separating pixels based on color intensity threshold.
(E) Clustering-based segmentation: Organizes food-related pixels into groups or clusters.
(F) Sobel operator-based segmentation: Infers food item edges by applying the Sobel operator to the image.
(G) Color and texture-based segmentation: Segments image based on color and texture characteristics.
(H) Thermal clustering: Distinguishes food from the plate and background using dynamic thermal thresholding.
(I) Region-based segmentation: Seeds are defined, then pixels meeting homogeneity criteria are added to the initial regions.
(J) CNN-based segmentation: Convolutional neural networks are used to localize food items, producing a binary image.

### 3.7. Volume and Nutrient Estimation

After food segmentation and classification, the next step is to estimate the portion size and compute the nutritional data of the food item. Volume can be estimated by multiplying the height and depth of the food [74]. Another method is based on pixel counting. In this method, each food group in the system has a predetermined nutritional value that is matched with a classification category. However, the cooking method may not always be this straightforward; for instance, the same dish may be fried or baked, which can result in radically different nutritional values [37]. Yet another strategy is to fit each food into a predetermined shape, e.g., a cylinder. The prism model, a volume estimation technique based on area, is used for food products without a clear three dimensional geometric structure. This food portion estimation method is based on a single-view food image, and is used to estimate the energy (in kilocalories) ingested during a meal. Utilizing geometric models, such as a container’s form, can aid in recovering some of the 3D properties of the food items in the scene. The volume of each food item in the picture may be calculated using the estimated 3D parameters of the item and a reference object in the scene. The food item’s density can then be used to estimate each food’s weight, for instance, an egg scrambled on a plate, with the size of the plate acting as a guide [75]. By fitting spherical or prismatic 3D models to the identified food regions, the food volume can then be determined. However, it is inevitable that using a single photograph to calculate the food’s volume will rely on frail assumptions about its three-dimensional shape. Certain systems have achieved improved findings by using several photos taken from different angles [7].

Another technique that is commonly used is three-dimensional (3D) model segmentation. The FoRConvD (Food Recognition using Convolutional Neural Networks and Depth Maps) method uses the phone camera alone to identify food categories and their mass on mobile devices. Depth map fusion is used to estimate the volume. In this technique, many photos are taken from different perspectives, then their depth maps are combined to create a 3D representation of the object. Experimental results have empirically demonstrated the accuracy and dependability of this food volume estimation approach, finding an overestimation of 0%–10% volume depending on the shape of the object [53]. Scale ambiguity is a concern when performing 3D reconstruction with a single smartphone camera. Comparing the dimensions of a reference object or fiducial marker with a known size and scale included in the photo beside the meal can help to determine food volume; examples include the user’s thumb or a coin, cup, bowl, or card [76,77]. To aid in calibrating measurements, the reference object or fiducial marker can also offer geometric data such as width/height [75,78,79]. Kong et al. used the multiple-view method. Their methods are simple and workable on common smart phones [40].

After applying the Adaboost feature selection method, Puri et al. applied dense stereo reconstruction. Despite the effectiveness of such reconstruction, the suggested method has drawbacks, requiring about half a minute for dense stereo reconstruction and volume estimation of a 1600 by 1200 pixel image [34]. To reach a faster processing time of 5.5 s per dish with a mean inaccuracy of less than 10% on 77 dishes of known volume, Dehais et al. suggested using two-view 3D reconstruction [17].

In a few cases, volume estimation has been performed using the user’s thumb as a reference in a two-dimensional image. Two images showing the user’s thumb and the plate are recorded from the top and side views [80]. To make it easier to estimate the area of various food shapes, another approach could be to superimpose a grid of squares over the top view image [81].

Chen et al. utilized a preconstructed shape model library of 3D geometric shapes to estimate meal volume. They compared the 3D shape model to the 2D meal outline to calculate the volume [82].

Macronutrients can be calculated or retrieved from a connected nutrition database if food types and weights are known. Accurate assessment of nutrients from food images relies on accurate measurement of the food volume. The first challenge in this process is to precisely identify each type of food that is contained in a dish and the cooking method used (e.g., blanching, baking, frying, or uncooked).

Situju et al. utilized a multitask CNN to assess the calorie and salt contents of food images in order to tackle cardiovascular disease. The dataset included salinity annotations for each image used to train the CNN [83].

It has been observed that systems using 3D modeling for food volume estimation provide better estimates than those based on single-view photos. Applications for vision-based dietary evaluation have proven to be reasonably accurate in calculating food quantities despite a number of significant difficulties mentioned in the literature, including view obstruction and scale ambiguity. Additionally, the suggested methods require that users capture many photographs of a single dish from different viewing angles, which is cumbersome for users and can be time-consuming. Depth sensing techniques for volume estimation have been presented as a remedy for view occlusion and scale ambiguity [84,85].

Overall, we find that the volume estimation phase can help diabetic patients/users to a great extent by helping them to calibrate food consumption according to healthy standards when managing their blood glucose. Furthermore, more accurate volume estimation in turn enhances the accuracy of blood glucose prediction for people with diabetes. Based on these data, they are able to make better dietary choices, be more aware, and have a healthier relationship with food. Lastly, these solutions can help to determine what kind of foods a patient is eating as well as how much of it they are consuming.

## 4. Findings

### 4.1. Challenges

Food picture identification has improved with deep learning methodologies; however, barriers still exist around recognizing multiple objects in images. Not every object is properly recognized, especially in photos with various foods or drinks. These results show that deep learning methods do not appear able to solve issues on their own without being integrated with other strategies. An effective diet assessment system should mention the type of food, ingredients, portion size, nutrients, etc. However, high variety and different appearance of foods in the same plate make this challenging. Different foods are frequently combined and cannot be optically separated, for instance, in a salad, soup, burger, fried rice, or pasta. Small variations in food products’ hue, shape, and texture can make segmentation difficult, especially in opaque substances; for example, the distinction between steamed and fried dumplings is barely perceptible. Many ingredients may not be visible in photos, while ingredients such as oil and fats are naturally opaque. It is essential to identify even opaque substances in order to accurately and usefully estimate the nutritional value of in a meal. The similarity between different foods’ appearances and the variability among the components that go into making different dishes, such as overlapping colors, ingredients, and shapes, makes the development of all-encompassing systems challenging.

Another issue is that multiple foods on the same plate may be occluded. Occlusion describes the information that is lost when an image is be captured from a certain angle due to objects being overshadowed by other objects, preventing flawless reconstruction of the 3D model. This unavoidably results in overestimation or underestimation of food volume.

In order to accurately compute 3D measures from 2D photos for volume estimation, several images are required. These treatments work best in controlled environments, and often cannot be utilized successfully in everyday situations, as users may find it inconvenient to capture multiple pictures of food from various perspectives. Thus, instead of being restricted to controlled environments, new solutions need to be investigated and tested in real-world day-to-day environments.

Customized food preparation methods of different users may also affect nutrient information. The absence of relevant labeled datasets and the impediment of extracting depth information from 2D images are additional difficulties that prevent high performance in the volume estimation stage. This process would be greatly streamlined if more mainstream mobile devices were to incorporate image depth acquisition sensors.

For proper food assessment, many photos taken from various angles are likely to correspond with higher accuracy. Despite being more user-friendly than multi-view techniques, single-view techniques can still be erroneous, as food consists of three-dimensional items. The majority of mobile devices come with RGB cameras instead of stereo depth-sensing cameras, limiting their ability to reveal all details of food’s 3D shape; for instance, the rear side of the dish is generally not visible. Typically, these photographs do not include any additional real-world details, such as scale, making volume estimation difficult. The inability to calculate daily macronutrient intake and its impact on blood glucose is a limitation of previous works, which have focused mostly on the classification of food items rather than determining their real volume and accompanying nutrient information.

Future FIRS solutions need to better address volume through recognition of food items that do not conform to simple shapes. The aforementioned algorithms have only been tested in existing research papers using modest model libraries made up of a variety of basic geometric shapes. The majority of the volume estimation techniques currently in use are only useful for solid and separable food products such as fruits and vegetables. We note that the greater part of current FIRS solutions do not include beverages. Beverages are frequently overlooked in FIRS, likely due to shape recognition difficulties; however, they should be included in diet assessments, as they contribute substantially to daily nutrient intake. For example, both dairy and non-dairy milk products provide carbohydrates, protein, and micronutrients that are associated with positive health outcomes. Alternatively, depending on the quantity consumed, sugar-sweetened beverages and those containing alcohol are associated with negative outcomes. Therefore, beverages should be included in datasets for dietary evaluation. However, due to their not possessing a distinct shape and possibly including intermingled components, drinks are more difficult to identify [86].

In light of its heavy dependency on the accuracy of earlier stages, such as food segmentation and volume estimation, macronutrient estimation continues to be a stage in automated diet assessment systems that is prone to inaccuracy [87]. As a result, the macronutrients may be overstated or underestimated if any of the previous phases are inaccurate.

We found that studies with larger datasets had more accurate categorization results. While we observed an increase in the number of datasets featuring foods from various cuisines, there is as yet no extensive training dataset featuring a wide variety of foods combining dishes from both eastern and western cuisines. Eastern cuisines generally have more varied cooking methods than western cuisines; therefore, it is necessary to create larger and more comprehensive datasets of food photos and to regularly update them using real-world images, perhaps through crowdsourcing. In addition, dishes within the same cuisine may vary due to differing ingredient measurements and cooking techniques.

Recognition and estimation of food volume, calories, and nutritional value via FIRS has thus far yielded encouraging results; however, more research is needed before it is ready for scaling up.

### 4.2. Future Research Directions

The most popular portable device for daily dietary/nutritional evaluation continues to be the smartphone [4]. Smartphone cameras, location systems, and inertial measurement sensors have the potential to provide rich multimodal data. However, current solutions are mostly limited to controlled environments such as laboratory settings, and have several practical constraints. Although there has been significant progress in developing automated and accurate FIRS and diet assessment systems in the academic world, end-user applications still depend mostly on manual recording of food types and portions. This emphasizes the necessity of more practical solutions that can be used in real-life situations. Therefore, it is vital to develop systems that can validate real-life usage scenarios and assess a variety of food images provided in various contexts using mobile consumer devices. In most cases, the user interacts regularly with the FIRS program to provide information regarding consumed food, such as a meal picture, meta-information, and weight progress, among others [9]. To boost user involvement and encourage interaction, methods such as scheduled reminders, notifications, or text messages asking the user to provide food photos or data can be employed in applications.

At the moment, FIRS projects are heavily data-driven and rely on dataset creation. Datasets containing photographs, ingredients, and other contextual information are required in order to ensure that algorithms can learn variations in the appearance of different foods. Furthermore, as there is no limit to the number of food items in a picture, food identification algorithms must be flexible in order to adjust to the constantly changing variants of food. However, the majority of datasets used in existing systems almost always exclusively include Western food.

The typical benchmark dataset should include a wide variety of culinary items from around the world [59]. Ideally, each item ought to have accompanying photos, a depth map, weight, nutritional data, a recipe, and a map of the distribution of the ingredients. In order to provide targeted solutions for diverse cuisines, it is necessary to develop an inclusive FIRS dataset with a variety of cuisine types. This will increase the use of FIRS and its popularity and, as deep learning primarily is data-driven, could speed up the development of improved FIRS algorithms. Massive quantities of tagged photos remain required for future technology and research.

More work should be done on putting together high-quality and inclusive labeled datasets that include the type of food, quantity, and nutritional values (carbohydrates, fat, sodium, etc.) of each item included in the training pictures. Considering that large food image datasets can enhance overall performance, a comprehensive and generic food image collection would boost benchmarking and performance evaluation. For instance, Min et al. demonstrated that their suggested large-scale dataset called Food2K performed well across a range of visual tasks, including food recognition and segmentation [88,89,90].

Using food recipes in the training phase can help to increase neural network performance. For instance, Fontanellaz et al. suggested that the initial stage in a pipeline for nutrient assessment could be multimodal recipe retrieval [91]. It is more scalable to understand food composition than to simply define food groups. Food recipes’ ingredient lists and cooking instructions can assist in better macronutrient estimation.

**Figure 7:**
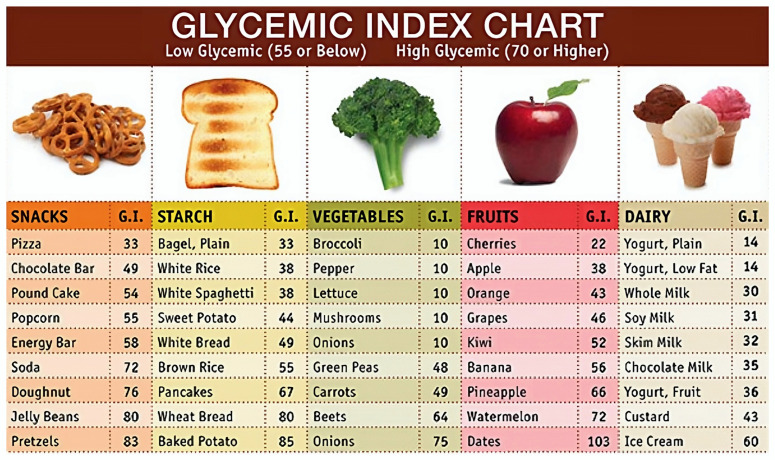
Glycemic index of selected common foods [93].

**Figure 8:**
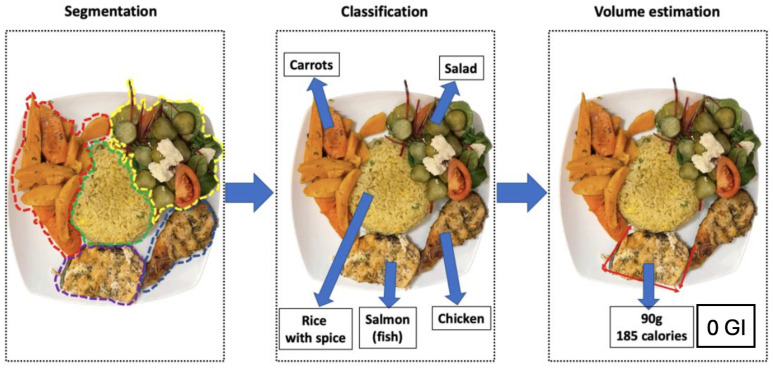
Schematic workflow of our proposed addition to current food image-based nutrition estimation methods (adapted from [14]). The glycemic index here is 0, as salmon does not contain any carbohydrates.

Diet analyses for monitoring blood glucose should focus on identifying the glycemic load of the individual meal. The glycemic index is a rating system for carbohydrate-containing foods. It refers to blood glucose changes that result from eating a certain quantity of a particular carbohydrate food while disregarding the influence of other nutrients. Figure 7 lists the GIs of selected foods. The glycemic load refers to the influence of the actual amount of that food eaten, i.e., one serving. However, integration of glycemic index and glycemic load works under the assumption that the food in question is eaten in isolation and not in combination with another food. In reality, most foods are eaten as part of a meal along with other items. In addition, certain foods such as proteins slow the digestion of carbohydrates, which can offset a high glycemic shift from a carbohydrate [92]. Future research should focus on integrating glycemic index/load prediction into these applications. Figure 8 depicts an ideal situation in food recognition, where the application recognizes the food and provides the GI value. These features have significant potential to positively impact the lives of and empower diabetic patients by alerting them to the potential effect of the meal that they are consumed on their blood glucose. Patients with diabetes can have their diet and glucose level monitoring automated, at least to some extent, by using this technology. Even though the estimations may not always be 100% accurate, they can help diabetic patients to assess and calibrate their diet in order to make it more healthy and diabetic-friendly.

Future generations of mobile devices may incorporate more sensors such as accelerometers and gyroscopes, as well as different kinds of cameras such as thermal and depth cameras, which could significantly increase the volume estimation capabilities of current nutrition monitoring applications. Applications may also provide users with the chance to contribute fresh crowdsourced training photos or cuisine categories to the training image library.

We believe that all of the above-mentioned studies demonstrate the considerable potential for translating image-based dietary/nutritional evaluation research prototypes into practical applications that can benefit diabetics as well as other medical groups.

## 5. Conclusions

This article provides a review of image-based nutritional monitoring methods and their potential for improving diabetes management applications. The correct recognition, segmentation, and volume estimation of food items are essential for accurate diet/nutrition assessment using FIRS. We have examined various techniques under development, with a focus on food classification and volume estimation methods. Convolutional neural networks are favored by researchers due to their high recognition accuracy. Combining CNNs with other methods can improve the accuracy of volume estimation for FIRS-based approaches, and we further suggest incorporating glycemic load to improve predictions of blood glucose impacts. More work is needed to improve the precision of these approaches, although it is clear that they have the potential to provide dietary recommendations to both users and clinicians that can result in improved health outcomes. FIRS-based suggestions can currently serve as a supplement for nutritionists/physicians as more research is conducted.

## Figures and Tables

**Table 1:** Inclusion and exclusion criteria.

Conditions for Inclusion	Conditions for Exclusion
The study must contain information about image recognition/classification in the field of nutrition, calorie, sugar, or blood glucose prediction	Studies focusing on other topics that do not include these keywords; only nutrition/calorie estimation, for instance
Paper is not a duplicate existing in a different database	Paper is already included, copy exists in a different database
Original research relevant to the topic in question	Similar studies or reviews in multiple scientific databases
Paper is written in English	Paper is not written in English
Peer–reviewed papers published in a conference proceeding or journal	Non peer–reviewed papers
Studies that have the full text available	Studies that don’t have full text available

**Table 2:** Food image recognition and/or nutrition evaluation publications specifically mentioning diabetes.

Author(s)	Application	Dataset	Methods	Accuracy	Year
Anthimopoulos et al. [6]	Food Recognition	≈5000 images, 11 classes	Bag-of-features (BoF)	78%	2014
Rhyner et al. [7]	Food Recognition/Carbohydrate Estimation (GoCARB)	Nine broad food classes—pasta, potatoes, breaded and non–breaded meat, rice, green salad/vegetables, mashed potatoes, carrots, and beans	Support Vector Machine	over 85%	2016
Usman et al. [8]	Testing Different DL Algorithms on Middle Eastern Food Recognition	7430 pictures under 38 different Middle Eastern food categories	Support Vector Machine	Individual Deep Models: AlexNet (57.46%), GoogleNet (59.15%), VggNet (59.45%) and ResNet (59.38%); Fusion Technique: PSO 66.16%, Equal Weights 65.38%, Early Fusion 62.22%	2019
Konstantakopoulos et al. [9]	Food Segmentation, Recognition and Volume Estimation (GlucoseML System)	Annotated Greek food images in the categories of: Dairy products or milk alternatives; Egg and its associated dishes; Meat and its associated dishes; Seafood and its associated dishes; Fats/Oils; Grain oand its associated dishes; Nut, seed or kernel based food; Vegetable and its associated dishes; Fruit and its associated dishes; Sugar and its associated dishes; and Miscellaneous foods	SIFT, SVM, ANN, SURF, RASNAC		2019
Alfonsi et al. [10]	Carbohydrate Counting Using Image Recognition (iSpy app)	200 common food images	CNN	94.5% (189/200)	2020
Sowah et al. [11]	Ghanaian Food Classification	300 images each in 25 classes	KNN (k = 5), Google’s ImageNet	over 95%	2020
Natephakdee et al. [12]	Thai Food Recognition	60,000 sample images	CNN	94.16%	2022

**Table 3:** Application of FIRS in dietary assessment steps.

Step	Action
1	User utilizes a smartphone camera to take a picture of the meal
2	Preprocessing takes place on image
3	Food varieties are separated from one another using segmentation procedures
4	Characteristics that are reliable and discriminative are extracted
5	The system’s subsequent phase receives input from the most crucial features that have been chosen
6	Food items are categorized into food categories
7	Each food item’s estimated volume is calculated
8	Utilizing reliable nutritional databases, the estimated calories and nutrients of the meal are shown
